# Student perceptions of medical improv in Sweden: an assessment of acceptability, relevance, and psychological safety

**DOI:** 10.1186/s12909-026-09614-9

**Published:** 2026-06-03

**Authors:** Lina Lantz, Inana Selenius, Jens Mellqvist, Oliver J Dyar

**Affiliations:** 1 Department of Public Health and Caring Sciences, Uppsala University, Uppsala, Sweden; 2 Teater Prego, Uppsala, Sweden

**Keywords:** Medical education, Applied improvisation, Culture

## Abstract

**Background:**

Medical improv is an innovative educational method that uses techniques and exercises from improvisation theatre to facilitate learning among healthcare students in a broad range of domains including communication, teamwork and professional skills. To date, there are few reports on the use of this pedagogical method outside of North America. We report here on the development, implementation and evaluation of a medical improv seminar series for undergraduate medical students in Sweden.

**Methods:**

We developed an extracurricular five-part seminar series in medical improv in collaboration with a local theatre group and according to best practice guidelines. The seminar series was held twice during spring 2024 and was open to all undergraduate medical students at Uppsala University. We developed a questionnaire to assess participating students’ perceptions of the acceptability and relevance of the seminar series, as well as experienced levels of psychological safety. Students voluntarily completed the questionnaire at the end of the fifth seminar.

**Results:**

Participating students (N = 17) were highly positive concerning the acceptability and relevance of the teaching method, and uniformly recommended the seminar series to fellow students. Our results provide strong evidence of high levels of experienced psychological safety in each participating student group at the end of each seminar series.

**Conclusions:**

Our study provides strong support for future efforts to implement medical improv as a teaching method in undergraduate settings and outside of North America. We contend that there are promising signs that medical improv may be a widely applicable teaching method that both stimulates learning in essential competency areas and fosters greater psychological safety; more broadly, it may provide a space for playfulness, stress-relief and the cultivation of resilience among medical students.

**Supplementary Information:**

The online version contains supplementary material available at 10.1186/s12909-026-09614-9.

## Background

Medical improv is an innovative educational method that uses exercises from improvisation theatre to facilitate learning among healthcare students in a broad range of domains including communication, teamwork and professional skills [1, 2]. A scoping review by Gao et al. suggested that medical improv can facilitate the acquisition of expertise in at least six of seven CanMEDS roles (medical expert, communicator, collaborator, professional, leader, and scholar) [1]. Teaching sessions typically consist of students engaging in exercises which each have a *focus* (i.e. a specific skill, such as receptive non-verbal skills), followed by a period of debriefing in which students reflect over the skills practiced in the exercise, the challenges encountered, and their relevance for clinical work as a doctor.

Several examples of implementations of medical improv within medical programmes have been described, and most often with favourable outcomes concerning knowledge and skills acquisition, and well-being [3–5]. These implementations have ranged from standalone seminars (typically voluntary, but in some cases compulsory) to elective courses, with varying degrees of integration into the medical programme [1, 6].

To date, the majority of implementations of medical improv in medical school contexts reported in the literature have been from North America. Thus, a core knowledge gap is the extent to which medical improv would be acceptable to and appreciated by medical students in other contexts where several relevant factors differ. For example, although general improvisation theatre courses and improvisation theatre performances have become more common in Sweden in recent years, improvisation theatre has not reached the heights of popularity as a cultural phenomenon as in the US. Medical improv as a pedagogical method, which is strongly inspired by improvisational theatre, may be associated with different preconceptions and levels of acceptance in Sweden. In addition, cultural factors can influence teachers’ and students’ perceptions of educational methods [7]. Finally, medical programmes in North America are solely postgraduate, in contrast to Sweden where most students begin their studies at one of the seven available programmes within three years of graduating high school, and without any previous university degree. Students at the early stages of the medical programmes in Sweden are thus typically younger than their North American counterparts, and are lacking (in relative terms) the academic and broader life experiences acquired through previous university studies.

After successful piloting of a standalone seminar for term two medical students at Uppsala University, Sweden during 2022, we subsequently developed and implemented a voluntary evening seminar series in medical improv together with a local theatre company. We aimed to assess participating students’ perceptions of the acceptability and relevance of medical improv through a written questionnaire at the end of the seminar series. Further, in line with recent publications suggesting the importance of creating a *safe environment* as part of medical improv [1, 4, 6], we evaluated students’ perceptions of *psychological safety* in the seminar series.

## Methods

### Development of the seminar series

In 2022, a study author (OD) developed a standalone seminar in medical improv at Uppsala University, together with a local theatre company. This voluntary seminar was offered as part of a regular course for term two medical students and received highly positive feedback in course evaluations. Supported by an educational grant from the medical programme, a seminar series consisting of five ninety-minute sessions was developed in 2023 together with a local theatre company and with representation from a final-year medical student throughout the development and implementation process. The series was developed according to identified best practices [1, 6], including:

- Establishing clear curriculum outcomes and ensuring exercises match the goals: each seminar series had a theme relevant to medical education, and the focus for each exercise matched the theme. The themes were: Communication; Collaboration; Body language; Failing well; Status and power.
- Establish trust and safety with appropriate sequencing of exercises: The first seminar began with an introduction to the pedagogical method, and a clear message that the primary focus of the seminar series was not on scene work or performing in front of an audience, but the development of core skills in communication and professionalism. We purposefully ordered the exercises within each seminar to create a safe environment for the students. We began each session with a simple, low demand, warm-up exercise as a whole group. We purposefully chose to include very few exercises in which there was any form of audience (i.e. students observing one another), preferring instead to have all students performing small group exercises simultaneously in parallel groups. The few exercises that were exceptions to this principle (i.e. exercises where there was some form of audience) were always scheduled towards the end of a seminar. In addition, the seminars were sequenced according to perceived complexity of the skills required, moving from communication skills (in which all students had previously had training in), to concluding with exercises around status and power dynamics (in which students were encouraged to incorporate their learning from the earlier seminars).
- Collaborate with experts in improvisation theatre: the seminar series was developed together with a local theatre company with long experience of applying improvisation techniques in non-theatre settings. Each seminar was *co-hosted* by a representative from the theatre company, a clinical teacher on the medical programme, and a final-year medical student.
- Choice of learning environment: the seminar series was conducted in a large, open room.
- Debriefing: many of the exercises were followed by a few short minutes of debriefing concerning the skills required and the challenges encountered, typically led by the representative from the local theatre company. Longer debriefing occurred typically at the middle and end of each seminar, in which students were encouraged to reflect over the relevance of the exercises and skills being trained to their medical studies and clinical experiences. These longer debriefs were supported by the clinical teacher and final-year medical student who also shared their own experiences from clinical settings.

Table 1 illustrates examples of exercises used in the five different seminars, as well as question prompts for reflections used during debriefing. A full list of exercises used in the seminar series is available on request from the corresponding author.

**Table 1:** Examples of exercises and reflection questions from the seminars

Theme	Example exercise	Example of reflection questions
1: Communication	Yes-and interview (in pairs): one student interviews the other on any topic of choice. The respondent replies to all questions with “Yes, and…”	How does our style of questioning influence the direction of a conversation? Have you encountered patients who tend to answer in the affirmative, irrespective of the question?
2: Collaboration	Robots (groups of 3–4): each group forms a line and walks around the room. The person at the back of the line controls the direction of the robot by sending signals (turn left, turn right, forwards, reverse, stop) along the line through tapping on shoulders. Students take turns to stand at the back.	Did anybody in the middle of the line feel a strong impulse to send a signal before they received one from the person behind them? How often were mistakes made, despite there being only five signals to send? How are mistakes made when we collaborate in clinical settings?
3: Body language	Body language in a circle (all stand in a circle): the group is given a short sentence (e.g. “I am angry at you” or “How nice to see you!”). One by one they take turns to say the sentence in as natural a possible a way to the next person in the circle. Then, using the same sentence, they one by one take turns to say the sentence in a way that their body language conflicts with the sentence.	How much variation was there in first example, where all said the sentence in a natural way for us? What feelings were awoken when you received a sentence with conflicting body language?
4: Failing well	Big fish, little fish (all stand in a circle): one student begins by sending a signal both verbally and with their hands to another student. The signal is either “Big fish” (illustrated by hands a short distance apart), or “Little fish” (illustrated by hands a long distance apart). If a student sends the signal incorrectly (e.g. “big fish” with hands far apart) then they leave the main circle and start a circle on the side where they continue to play the same exercise as new students join from the initial circle after also making mistakes.	What feelings arose when you made a mistake? How do these feelings compare with when you make a mistake in your medical studies, or in a clinical setting? When your clinical supervisors have made mistakes, how have they reacted?
5: Status	Status swap scenes (in pairs): students play a scene (e.g. job interview) where one person begins the scene with a high status and the other with a low status. After a couple of minutes, when the leader claps, the students need to gradually swap status levels in the scene, whilst maintaining their respective roles and the narrative of the scene	What methods did you use to express your status during the scenes? Was there any connection between your status and your mood in the scene? How do you experience that status is expressed in clinical settings? Do your supervisors attempt to “give” you status in front of colleagues, patients? If yes, how?

### Participants and implementation

Medical students from all eleven terms at Uppsala University were eligible to attend the seminar series, although a maximum class size of fifteen students was decided upon in advance, based on previous experiences of medical improv. The medical programme in Uppsala University is broadly divided into a pre-clinical studies stage (terms 1 to 4 [year 1–2], with a primary focus on basic sciences, professional development and some early clinical contact at primary care practices to develop basic clinical skills in history taking and examination), and a clinical studies stage (terms 5 to 11 [year 3-5.5], based primarily at hospitals, and with additional time in primary care in the final term). At any timepoint there are roughly 100 students studying each term, since there is both spring and autumn intake to the medical programme.

Two iterations of the seminar series were offered during the 2024 spring semester, with only very minor changes made between the two iterations (e.g. removal of one exercise which had performed less well; tweaking of the formulation of certain reflection prompts). The seminar series was advertised to students using posters, social media and an announcement on the programme’s digital learning platform. Attendance at all five sessions in a seminar series was encouraged but was not a requirement. Each session in the seminar series lasted ninety minutes and was conducted in Swedish.

### Evaluation

A written questionnaire [Supplementary material 1] was developed by the co-authors to assess participating students’ perceptions of the medical improv seminar series. Questions were developed after a review of the literature with a focus on acceptance and relevance to medical studies (ten questions), and experienced psychological safety (seven questions), the latter of which were based on Edmondson’s classic psychological safety questionnaire [8], translated into Swedish and with very minor adaptations. All questions were answered using a seven-point Likert scale (1 = completely disagree, 7 = completely agree) and students were informed that questions could be skipped if they preferred not to answer. The questionnaire was available for students to complete at the end of the fifth seminar, and participating was both voluntary and anonymous.

## Results

### Participation

A total of twenty-one students attended the first session of one of the two seminar series, with seventeen students participating in at least three of the sessions in a series. All seventeen students responded to the questionnaire. Seven of these students were currently attending pre-clinical courses (terms 1–4) and ten were attending clinical courses (terms 5–11).

### Acceptability and relevance

The students perceived the seminar series to be highly acceptable and relevant to their medical studies, with each sub-domain receiving a mean between 6 and 7 on the seven-point scale (Table 2). All students reported that they would recommend the course to fellow medical students.

**Table 2:** Acceptability and relevance of medical improv seminar series (*N* = 17)

Question	Mean score* [standard deviation]	Agree with statement (%)
The seminar series was fun	6.9 [0.33]	100
The seminar series was relevant to me as a medical student	5.9 [1.39]	88.2
The seminar series gave me insight into my strengths and weaknesses in collaboration	6.0 [1.17]	94.1
The seminar series gave me insight into my strengths and weaknesses in communication	5.8 [1.38]	82.4
The seminar series strengthened my self-esteem	6.1 [0.86]	100
I can use lessons from the seminar series in my medical studies	6.5 [0.72]	100
The seminar series gave me valuable insights into various aspects of the medical role	5.9 [0.86]	94.1
The seminar series complements the teaching in professional development on the medical program	6.5 [0.62]	100
I would recommend the seminar series to other medical students	6.9 [0.33]	100
The seminar series is suitable to be completed with students from other healthcare professions	6.9 [0.33]	100

*On a seven-point Likert scale where 1 = completely disagree, and 7 = completely agree

### Psychological safety

Students’ responses to questions concerning psychological safety indicate a high level of experienced psychological safety in the group at the end of the seminar series (Table 3).

**Table 3:** Experienced psychological safety of medical improv seminar series (*N* = 17)

Question	Mean score* [standard deviation]
If you made a mistake during the seminar series, it was often held against you	1 [0]
Seminar series participants could discuss problems and difficult topics	6.1 [1.65]
It happened that seminar series participants or course leaders excluded others because they were different	1 [0]
It was safe to take risks during the seminar series	6.2 [1.59]
It was difficult to ask other seminar series participants or course leaders about help	1.2 [0.44]
No one intentionally acted in a way that undermined my efforts (unless that was the purpose of a specific exercise)	4.8 [2.92]
My unique skills and talents were valued and utilized during the seminar series	5.5 [1.63]

*On a seven-point Likert scale where 1 = completely disagree, and 7 = completely agree

### Variations between respondents

Results according to subgroups (preclinical students vs. clinical students; participating in the first seminar series vs. second seminar series) are included in Supplementary Table 1 (acceptability and relevance) and Supplementary Table 2 (psychological safety). No statistical tests were conducted to formally compare subgroups due to the limited number of participants in each subgroup, but the descriptive results (means) indicate that there were only extremely minor differences between the different subgroups.

## Discussion

We developed a five-part seminar series in medical improv open to all medical students at Uppsala University, Sweden, and our questionnaire-based evaluation data provide the first insights on an implementation of medical improv outside of North America and in an undergraduate context.

Similar to previous reports [4, 9], participating students were highly positive concerning the acceptability and relevance of medical improv as a teaching method. Students uniformly stated that they would recommend participating in the seminar series to their fellow students, and we found no evidence of meaningful variations between preclinical (term 1–4) and clinical (term 5–12) students, although it must be cautioned that our small number of participants limited the ability of formal statistical testing for differences. Future studies with larger sample sizes may be more capable of identifying differences between different subgroups (e.g. stage of medical education, gender, age, and other demographic factors). Our findings nevertheless suggest that the developed content (i.e. exercises) and the debriefing discussions during the seminars are considered valuable for the students, irrespective of how far they have progressed in their medical studies. This is perhaps somewhat surprising since specific teaching sessions using other teaching methods may be considered most relevant at specific time points in a medical programme (for instance, due to the subject requiring the acquisition of knowledge from earlier courses, or the inherent difficulty level of the topic). Informally, the study authors observed that the debriefing discussions were very naturally balanced in terms of participation throughout the seminar series, with no individual or group (e.g. students from the same course) dominating, and students appearing to appreciate and build upon one another’s oral reflections, irrespective of which term they belonged to.

Psychological safety was recently described as a prerequisite for optimal learning in medical education by McClintock et al. [10] in a scoping review, “allowing students to be present in the moment, focus on the task at hand, and ask questions without fear of negative interpersonal consequences”. The same authors concluded, however, that the available data point to a lack of psychological safety in medical education. Our results provide strong evidence of high levels of psychological safety in each group of the two student groups at the end of the two iterations of the seminar series in medical improv. This was despite students coming from a variety of terms across the medical programme and with few pre-existing friendships (data not shown). This finding is in keeping both with a previous report of Li et al. [4] in which participants described a medical improv workshop as helping to build psychosocial skills in a *safe environment*, and with broader recommendations for medical improv that emphasise the importance of a *culture of safety* [1, 6]. As described in the methods, we intentionally put much preparation before the seminar series was implemented into establishing trust and safety, in part through clear messaging about the content and purpose of the seminar series (i.e. not performance-oriented, but skills development), as well as the choice and sequencing of the exercises both within each session as well as the series as a whole. We contend that this groundwork is likely to have contributed to the positive outcomes in terms of experienced psychological safety.

Our study provides strong support for future efforts to implement medical improv as a teaching method in undergraduate settings and outside of North America, although it is important to recognize that our findings are based on a small number of participants who voluntarily participated in the seminar series. Risks exist for selection biases of individuals who were most open to alternative teaching forms, although only one participating student reported having previous experience with improvisation theatre. Four students participated in the first session of either of the two iterations of the seminar series and chose not to participate in further sessions; importantly, we do not have any information on their reasons for subsequent non-attendance, so it is not possible to rule out low perceived relevance or low experienced psychological safety for these individuals as a reason, even if our informal observations of these particular students’ engagement in the initial sessions did not given any indication of such a rationale. Despite these caveats, the primary outcomes of this study are at least informally strengthened by the subsequent experiences of the senior author with implementing a seminar in medical improv for term 7 medical students, as part of a course in communication skills and leadership. This seminar is obligatory for all students to attend, uses several of the exercises trialled during the seminar series, and has received very positive feedback from participants over the past four terms in course evaluations.

Potential avenues for future research include the development of tools to more objectively assess learning outcomes and their long-term stability, as well as techniques for assessing how experienced levels of psychological safety may develop and fluctuate during a seminar series. These can help overcome the limitations in our study (and similar previous studies) of using non-validated questions to assess relevance and acceptance, as well as relying on more subjective measures of self-reported skill development. We found value in applying Edmondson’s questionnaire but experienced that the question concerning valuing an individual’s unique skills and talents was perhaps less directly relevant in this group-centred educational context. In addition, due to the nature of our exercises which included a focus on failure, we found it challenging to formulate the item “No one intentionally acted in a way that undermined my efforts”; we thus added a suffix “(unless that was the purpose of a specific exercise)” to the item, but our pattern of responses suggest that the phrasing may have been misinterpreted: five respondents indicated they strongly disagreed (1), and all but one other indicated they strongly agreed (7). No other item yielded such divergent responses. 

## Conclusion

Finally, in light of the growing concerns worldwide over medical student mental health and well-being [11], we conclude that there are promising signs from our present study, our current seminars on the programme in Uppsala, and the broader growing literature base, that medical improv may be a widely applicable teaching method capable of both stimulating learning in essential competency areas whilst also contributing to greater psychological safety – and more broadly, a space for playfulness, stress-relief and the cultivation of resilience among medical students [1, 12].

## Supplementary Information

Supplementary Material 1.

Supplementary Material 2.

## Data Availability

The datasets used during the current study are available from the corresponding author on reasonable request.
