# Supplementary material for: Student perceptions of medical improv in Sweden: an assessment of acceptability, relevance, and psychological safety
Source: BMC Med Educ. 2026 Jun 3;26:933. doi: 10.1186/s12909-026-09614-9 (PMC13242137; doi:10.1186/s12909-026-09614-9)
Supplement: Supplementary file 1 — Supplementary Material 1. [file 12909_2026_9614_MOESM1_ESM.pdf]

Evaluation Medical Improv

The survey will be used to evaluate and develop the seminar series.

The survey is completely anonymous and voluntary.

Term: T1-T4 ☐ T5-T11 ☐

To what extent do you agree with the following? 1 - not at all, 7 - completely agree							
**Statement**	**Grading**						
1. The course was fun	1 ☐	2 ☐	3 ☐	4 ☐	5 ☐	6 ☐	7 ☐
2. The course was relevant to me as a medical student	1 ☐	2 ☐	3 ☐	4 ☐	5 ☐	6 ☐	7 ☐
3. The course provided insight into my strengths and weaknesses in teamwork	1 ☐	2 ☐	3 ☐	4 ☐	5 ☐	6 ☐	7 ☐
4. The course provided insight into my strengths and weaknesses in communication	1 ☐	2 ☐	3 ☐	4 ☐	5 ☐	6 ☐	7 ☐
5. The course boosted my confidence	1 ☐	2 ☐	3 ☐	4 ☐	5 ☐	6 ☐	7 ☐
6. I can apply the lessons from the course in my medical studies	1 ☐	2 ☐	3 ☐	4 ☐	5 ☐	6 ☐	7 ☐
7. The course gave me valuable insights into various aspects of the doctor’s role	1 ☐	2 ☐	3 ☐	4 ☐	5 ☐	6 ☐	7 ☐
8. The seminar series complements the existing course on professional development in the medicine programme	1 ☐	2 ☐	3 ☐	4 ☐	5 ☐	6 ☐	7 ☐
9. I would recommend the course to other medical students	1 ☐	2 ☐	3 ☐	4 ☐	5 ☐	6 ☐	7 ☐
10. The course is suitable to conduct with students from other healthcare professions	1 ☐	2 ☐	3 ☐	4 ☐	5 ☐	6 ☐	7 ☐

Evaluation Medical Improv

To what extent do you agree with the following? 1 - not at all, 7 - completely agree							
**Statement**	**Grading**						
1. If you made a mistake during the course, it was often held against you	1 ☐	2 ☐	3 ☐	4 ☐	5 ☐	6 ☐	7 ☐
2. Course participants were able to bring up problems and tough issues	1 ☐	2 ☐	3 ☐	4 ☐	5 ☐	6 ☐	7 ☐
3. There were instances where course participants or instructors rejected others for being different	1 ☐	2 ☐	3 ☐	4 ☐	5 ☐	6 ☐	7 ☐
4. It was safe to take risks during the course	1 ☐	2 ☐	3 ☐	4 ☐	5 ☐	6 ☐	7 ☐
5. It was difficult to ask other participants or instructors for help	1 ☐	2 ☐	3 ☐	4 ☐	5 ☐	6 ☐	7 ☐
6. No one deliberately acted in a way that undermined my efforts (if it was not the purpose of the exercise)	1 ☐	2 ☐	3 ☐	4 ☐	5 ☐	6 ☐	7 ☐
7. During the course, my unique skills and talents were valued and utilized	1 ☐	2 ☐	3 ☐	4 ☐	5 ☐	6 ☐	7 ☐

Possible improvements and other comments

Are there any skills that you wish we had covered? How can we make the course better? Is there anything else you would like to mention?
