# Supplementary material for: Student perceptions of medical improv in Sweden: an assessment of acceptability, relevance, and psychological safety
Source: BMC Med Educ. 2026 Jun 3;26:933. doi: 10.1186/s12909-026-09614-9 (PMC13242137; doi:10.1186/s12909-026-09614-9)
Supplement: Supplementary file 2 — Supplementary Material 2. [file 12909_2026_9614_MOESM2_ESM.docx]

**Supplementary Table 1. Acceptability and relevance of medical improv seminar series – by subgroup**

| Question |  | Mean score* | |  |
| --- | --- | --- | --- | --- |
|  | Term 1-4, pre-clinical  (n = 7) | Term 5-11, clinical  (n = 10) | First iteration  (n = 9) | Second iteration  (n = 8) |
| The seminar series was fun | 7 | 6.9 | 6.8 | 7 |
| The seminar series was relevant to me as a medical student | 5.5 | 6.2 | 5.7 | 6.3 |
| The seminar series gave me insight into my strengths and weaknesses in collaboration | 5.8 | 6.2 | 5.9 | 6.1 |
| The seminar series gave me insight into my strengths and weaknesses in communication | 6.2 | 5.6 | 5.8 | 5.9 |
| The seminar series strengthened my self-esteem | 6.2 | 6.1 | 6.2 | 6 |
| I can use lessons from the seminar series in my medical studies | 6.3 | 6.5 | 6.6 | 6.4 |
| The seminar series gave me valuable insights into various aspects of the medical role | 5.5 | 6.1 | 5.9 | 5.9 |
| The seminar series complements the teaching in professional development on the medical program | 6.3 | 6.7 | 6.7 | 6.4 |
| I would recommend the seminar series to other medical students | 6.8 | 7 | 6.9 | 6.9 |
| The seminar series is suitable to be completed with students from other healthcare professions | 6.8 | 6.9 | 6.9 | 6.9 |

*On a seven-point Likert scale where 1 = completely disagree, and 7 = completely agree.

**Supplementary Table 2. Experienced psychological safety of medical improv seminar series – by subgroup**

| Question |  | Mean score* | |  |
| --- | --- | --- | --- | --- |
|  | Term 1-4, pre-clinical  (n = 7) | Term 5-11, clinical  (n = 10) | First iteration  (n = 9) | Second iteration  (n = 8) |
| If you made a mistake during the seminar series, it was often held against you | 1 | 1 | 1 | 1 |
| Seminar series participants could discuss problems and difficult topics | 6 | 6.6 | 6.1 | 6.1 |
| It happened that seminar series participants or course leaders excluded others because they were different | 1 | 1 | 1 | 1 |
| It was safe to take risks during the seminar series | 5.7 | 6.7 | 6.4 | 5.9 |
| It was difficult to ask other seminar series participants or course leaders about help | 1.2 | 1.3 | 1.1 | 1.4 |
| No one intentionally acted in a way that undermined my efforts (unless that was the purpose of a specific exercise) | 4.8 | 5.2 | 4.9 | 4.8 |
| My unique skills and talents were valued and utilized during the seminar series | 5.2 | 5.6 | 6 | 5 |

*On a seven-point Likert scale where 1 = completely disagree, and 7 = completely agree.
